# Critically appraised topic on adverse food reactions of companion animals (2): common food allergen sources in dogs and cats

**DOI:** 10.1186/s12917-016-0633-8

**Published:** 2016-01-12

**Authors:** Ralf S. Mueller, Thierry Olivry, Pascal Prélaud

**Affiliations:** Medizinische Kleintierklinik, Centre for Clinical Veterinary Medicine, Ludwig Maximilian University, Munich, Germany; Department of Clinical Sciences, College of Veterinary Medicine, North Carolina State University, Raleigh, NC USA; Clinique Advetia, Paris, France

**Keywords:** Allergen, Allergy, Atopic dermatitis, Canine, Cat, Dietary, Dog, Feline, Food allergy

## Abstract

**Background:**

To diagnose cutaneous adverse food reactions (CAFRs) in dogs and cats, dietary restriction-provocation trials are performed. Knowing the most common offending food allergens for these species would help determining the order of food challenges to optimize the time to diagnosis.

**Results:**

The search for, and review and analysis of the best evidence available as of January 16, 2015 suggests that the most likely food allergens contributing to canine CAFRs are beef, dairy products, chicken, and wheat. The most common food allergens in cats are beef, fish and chicken.

**Conclusions:**

In dogs and cats, after a period of dietary restriction leading to the complete remission of clinical signs, food challenges to diagnose CAFR should begin with beef and dairy products, the most commonly recognized food allergens in these two species.

## Background

The diagnosis of cutaneous adverse food reactions (CAFRs) in dogs and cats relies on the performance of dietary restriction-provocation trials. Knowing the most common offending allergens in these species would help determine which food challenges should be performed first to faster confirm the diagnosis of CAFR.

**Table 1:** Details of studies about allergens suspected of causing CAFRs in cats

Reference	Number of dogs	Number of individual rechallenges per dog	Details of rechallenges	Offending allergens and comments
Walton [2]	82	unclear	unclear	cow's milk (22), tinned food (16), beef (13), wheat (11), mutton (6), egg, pork (3 each), herring (2), cod, maize, rabbit, dog biscuits, kidney bean (1 each)
Chesney [4]	19	unclear, but only 9 owners rechallenged their dogs	unclear	beef (4), milk (3), chicken, dog biscuit (2 each), cheese, turkey, pork (1 each)
Guilford et al. [5]	8	3 to 6	rechallenge with corn, soy, cow's milk (8/8 each), wheat (2/8), lamb (7/8), beef (3/8) (14-16 g of each, once daily for 14 days)	corn (2), wheat, milk (1 each)
Harvey [7]	25	at least 6	1 week of beef, milk, cheese, egg, mixer biscuit, bread in all dogs, additionally chicken, lamb and chocolate one each in 3 of the dogs	bread and mixer biscuit (same 7 dogs), cheese and milk (same 7 dogs), beef (2), egg, lamb, chocolate (1 each)
Ishida et al. [8]	8	9	beef, chicken, chicken egg, wheat, corn, rice, tuna, cod, milk (increasing to 120 g daily for 7 days)	beef (5), rice (3), egg, wheat, cod (1 each)
Jeffers et al. [9]	13	6	beef, cow's milk, chicken, chicken egg, soybean, wheat for 1 week each	beef (12), milk (5), wheat (4), chicken, soybean (3 each), egg (2)
Jeffers et al. [10]	25	5	beef, chicken, chicken eggs, cow's milk, soy for 1 week each	beef (15), soy (8), chicken, milk (7 each), wheat (6), 5 (egg)
Mueller and Tsohalis [11]	8	unclear	unclear	beef (7), dairy (1), chicken (1)
Ohmori et al. [12]	1	unclear	unclear	beef
Paterson [13]	20	at least 5	Beef, dairy products, wheat, lamb and chicken for one week each as one additional test meal while on elimination diet followed by other allergens based on diet history	beef (13), lamb (5), gluten, egg (4 each), chicken, milk, pork (2 each), soy, corn (1 each)
Salzo [16]	20	unclear	7 days of rechallenge	beef (11), chicken (7), rice (5), milk, wheat (1 each)
Vaden et al. [17]	6	7	1 meal containing a different allergen every other day	chicken, corn (5 each), tofu (3), cottage cheese, wheat, lamb (2)
Coyner [18]	1	1	unclear	beef
Mueller et al. [20]	1	5	rechallenge with beef, cow's milk, chicken, mutton, wheat, details unclear	beef
Nicholls et al. [21]	2	unclear	unclear	beef (2)
Johansen et al. [23]	4	unclear	unclear	beef (2), pork, salmon (1 each)
Johansen et al. [24]	5	unclear	unclear	chicken (5), corn (3), but preselected out of a population with such allergies
Tarpataki and Nagy [25]	39	unclear	unclear	chicken (16), beef (12)
Fujimura et al. [26]	1	1	10 g of fresh tomato led to clinical signs within 30 minutes	tomato

**Table 2:** Details of studies about allergens suspected of causing CAFRs in cats

Reference	Number of cats	Number of individual rechallenges per cat	Details of rechallenges	Offending allergens and comments
Stogdale [1]	2	6 in 1 cat, unclear in the other	Chicken, fish, beef, horse, mutton, milk in one cat, various fresh meats and commercial foods in the other cat, details unclear	chicken, fish, beef in the first cat, and not chicken and fish in the other cat (all other meats led to deterioration)
Walton [2]	18	unclear	unclear	cow's milk (7), beef (5), rabbit, chicken, whale meat (1 each)
White and Sequoia [3]	14	unclear	various commercial diets, dairy products, fish were administered to 11 of 14 cats further details were not provided	fish (6), dairy products (2)
Guilford et al. [6]	16	unclear, depending on the diet history	15-50 g of allergen daily for 7 days	beef, corn, wheat (3 each), gluten, barley, chicken, lamb, sardines, lactose, viscera, food additives (1 each)
Reedy [14]	1	2	3 days of tuna and lamb	tuna, lamb (1)
Guaguère [19]	10	unclear	2 weeks with each allergen	beef (4), milk (3), fish (2), egg (1)
Walton et al. [22]	1	unclear	7 days with each allergen	milk
Vogelnest and Cheng [27]	17		beef, chicken, lamb, fish, diary, wheat for 7 days each (attempted only in 8 cats and completed in 6 of them)	fish (2), chicken (1), beef (1)

### Clinical scenarios

You have two patients: The first is a 1-year-old male Labrador retriever with a 3-month history of pruritus and recurrent mucous diarrhea. This dog has been eating a commercial diet for the last 6 months. On physical examination, you do not detect anomalies besides soft stools on rectal palpation. Your second patient is a 2-year-old female spayed Persian cat that has been scratching her face for the last year. This self-trauma only responds partially to high dose of prednisolone. Physical examination reveals the cat to be thinner than expected and to have excoriations on the head and neck. You suspect that both patients could be reactive to their commercial diets, but you wonder which one of the ingredients listed on the labels would be the most likely sources of allergens.

### Structured question

In dogs and cats suspected of CAFR, which food sources are most often reported to induce clinical signs after challenge?

### Search strategy

The CAB Abstracts and Web of Science (Science Citation Index Expanded) databases were searched on January 16, 2015, using the following string: ((dog or dogs or canine) or (cat or cats or feline)) and (food or diet*) and (allerg* or atop* or hypersens* or intolerance). The search was limited to the period 1985 to 2015. Bibliographies of identified articles were then further searched for additional relevant reports.

### Identified evidence

Our literature search identified 140 and 1534 citations in CAB Abstracts and Web of Science, of which three [1–3] and 15 [1, 3–17] respectively contained relevant information. Citations that were not selected were those of articles not specifically identifying offending allergens in dogs and cats exhibiting clinical signs of CAFR. Six more relevant citations were identified in the bibliography of articles found with the electronic search [18–22], and three sources were abstracts of recent conference proceedings [23–25]. Offending allergens were reported in case reports [12, 14, 18, 22, 26] or case series of dogs and cats with clinical evidence of adverse food reaction [1–5, 7, 10, 13, 16, 19, 25, 27], in studies evaluating diagnostic techniques for adverse food reactions [5, 8, 9, 11, 17, 23, 24] or (rarely) in studies evaluating reaction patterns such as vasculitis or symmetrical lupoid onychitis with multiple causes [20, 21]. A positive rechallenge was considered the only solid evidence for identifying an offending allergen. From these selected publications, we added the number of cases in which positive challenges had occurred with the various food items, and the frequency of reaction among total number of dogs was calculated.

### Evaluation of evidence

Altogether, at least one offending food allergen source was reported in each of the 297 dogs included in the selected studies [2, 4, 5, 7–13, 16–18, 20, 21, 23–26] (Table 1). The most frequently reported food allergens involved in CAFRs in dogs were beef (102 dogs, 34 %), dairy products (51 dogs, 17 %), chicken (45 dogs, 15 %), wheat (38 dogs, 13 %) and lamb (14, 5 %). Other less commonly reported offending food sources were soy (18 dogs, 6 %), corn (13 dogs, 4 %), egg (11 dogs, 4 %), pork (7 dogs, 2 %), fish and rice (5 dogs each, 2 %). Barley, rabbit, chocolate, kidney bean and tomato were also reported as food allergens for single dogs.

At least one food allergen was identified in each one of the 78 cats reported in selected articles [1–3, 6, 14, 19, 22, 27] (Table 2) . The food sources most frequently causing CAFR in cats were beef (14 cats, 18 %), fish (13 cats, 17 %), chicken (4 cats, 5 %), wheat, corn and dairy products (3 cats each, 4 %) and lamb (2 cats, 3 %). Egg, barley and rabbit were also reported as offending allergens in individual cats.

There were several limitations in interpreting the data presented. In most studies details of the provocation with individual allergens were not provided. Furthermore, most reports only listed allergens associated with a deterioration of signs upon rechallenge, but not those associated with negative provocations; this could possibly bias the estimation of the prevalence of offending allergens. Only five studies had used a standardized rechallenge sequence in dogs [7–10, 13]. In these studies, beef, chicken, wheat, soy and dairy products were the most common involved allergens, reflecting the data gathered from the literature. In cats, only one study attempted those uniform provocations [27], and beef, fish and chicken were the allergens most commonly involved in that study. In addition the previous diet history was generally not provided, thereby preventing a clinically relevant interpretation of the data. Thus, the information gathered herein does not allow a true estimate of the prevalence of offending allergens nor any statement about the likelihood of positive provocations in relation to previously fed foods. Finally, the offending allergens found herein could merely reflect pet feeding habits in the preceding decades, and these allergens could change once new pet foods become fashionable and used more frequently.

### Conclusion and implication for practitioners

In a dog living in Australia, Europe or North America, the allergens most likely contributing to CAFRs are beef, dairy products, chicken, wheat and lamb. As a result, these foods should be the first used for allergen provocation for CAFR diagnosis. In cats, the most common allergens causing CAFRs are beef, fish and chicken.

Importantly, the identified evidence does not allow an estimation of the real prevalence of offending allergens in the population of dogs and cats with CAFR, as animals were usually only challenged with a small number of—but not all— allergens. As a result, the true prevalence of each offending allergens in dogs and cats is likely to be higher than that reported above.

Importantly, all these estimates of prevalence will need to be reevaluated with prospective studies performing controlled rechallenges in a larger number of animals with a detailed history of their previous dietary exposure.

## Abbreviations

CAFR: cutaneous adverse food reaction
CAT: critically-appraised topic
